# A Cross-Sectional Study on the Application of IS in Perioperative Pulmonary Function Training in Spine and Orthopedics

**DOI:** 10.1155/2022/4546549

**Published:** 2022-07-06

**Authors:** Ting Qiu, Yong Li, Jingjing Zhang, Xuanzhu Hou, Yuqi Wu, Yan Xu, Wenyue Chen, Jingjing Rui, Jin Yang, Jing Qian

**Affiliations:** Infection Control Administration Department, The Affiliated Hospital of Nanjing University Medical School, Nanjing 210008, China

## Abstract

**Background:**

The incentive spirometer (IS) is a mechanical device that promotes lung expansion and can be used to prevent and treat postoperative pulmonary complications. In this study, the preventive effect of pulmonary function training with IS on the improvement of pulmonary function and pulmonary complications was observed.

**Methods:**

From May 2019 to April 2021, 69 scoliosis patients with impaired moderate and severe lung function were divided into the traditional pulmonary training group (*n* = 32) and IS-enhanced pulmonary training group (*n* = 35). The patient underwent lung function testing after admission and one week after the start of training and chest CT on the third day after surgery.

**Results:**

The average age was 13.47 and 15.66, respectively (*p* = 0.223). The Cobb angles were 83.84 and 83.97 (*p* = 0.756), respectively, and no statistical difference between the parameters of lung function was detected. After 1 week of respiratory function training, significant improvement in lung function testing parameters including VC%, FVC%, FEV1%, FEV1/FVC, FEV1/VC, and MVV% was found in both groups. Analysis of covariance showed more significant improvement in IS-enhanced group compared to the conventional training group (*p* < 0.05). The incidence of postoperative pulmonary atelectasis was lower in IS-enhanced group than in traditional groups (2.9% vs. 21.9%, *p* = 0.043) with no difference in the overall incidence of pulmonary complications (*p* = 0.164) and shorter preoperative and total hospitalization in the IS-enhanced group.

**Conclusion:**

Compared to traditional pulmonary function training, IS-enhanced training can significantly accelerate the improvement of pulmonary function testing parameters, shorten the preoperative pulmonary function training time, reduce the incidence of postoperative pulmonary tension complications, and accelerate postoperative rehabilitation.

## 1. Introduction

Surgery is the main treatment for scoliosis. As one of the most important physiological dysfunction caused by scoliosis, impaired lung function is an important factor affecting the safety of surgical indications and the perioperative period. Studies have shown that impaired lung function can induce respiratory failure and even death [1]. It is extremely important to improve lung function and its compensatory ability by preoperative pulmonary function training for reducing postoperative lung complications and improving the safety of surgery. Traditional methods of preoperative respiratory rehabilitation training have been shown to have a significant effect on improving the patient's respiratory function, including lip compression, balloon blowing, breathing exercises, etc. The disadvantage is that the training process lacks clear quality control indicators and is difficult to self-assessment. This depends heavily on the patient's understanding and compliance, thus significantly reducing the efficiency of training.

Unlike traditional lung function training methods, the incentive spirometer (IS) is a mechanical device to assist lung dilation, which helps patients keep their lungs in good condition by increasing the total lung capacity (TLC) and lung compliance, improving oxygen supply, and maintaining the smoothness of the small airways. IS provides clear feedback during breathing training, which enables patients to set and achieve lung function training goals. Studies have shown that IS can effectively improve a patient's breathing function during the perioperative period of chest and abdomen surgery, which improves surgical tolerance and reduces the occurrence of postoperative lung complications. IS is also widely used in the scoliosis orthopedic perioperative period, but its actual role in the improvement of perioperative lung function is lack of a clear evaluation.

The purpose of this study is to evaluate the role of IS in the training of pulmonary function during scoliosis orthopedic perioperative surgery and its effect on postoperative complications.

## 2. Information and Methods

### 2.1. Subjects

Patients undergoing scoliosis orthopedic surgery at the Spinal Surgery Center of Drum Tower Hospital, affiliated with Nanjing University Medical College, from May 2019 to May 2021, whether patients who had normal communicational skills and cognition, and had no correlative surgical treatment history, were enrolled.

#### 2.1.1. Inclusion Criteria

Inclusion criteria include the following: (1) malformations affecting congenital or recurrent scoliosis of the chest; (2) Cobb angle: 50–100°; (3) age: 12–18 years; with the completion of lung function examination and respiratory function training program, gender is unlimited; and (4) the lung function report shows moderate and severe pulmonary dysfunction.

#### 2.1.2. Exclusion Criteria

Exclusion criteria include the following: (1) patients with simple lumbar scoliosis and an upper lateral terminal cone below the L1 segment; (2) patients who combine other diseases and are unable to cooperate with examination and training; (3) patients with severe heart, liver, and kidney dysfunction; (4) blood gas analysis at the time of admission showed severe respiratory failure; and (5) patients combined with other underlying lung diseases.

#### 2.1.3. Remove Criteria

Remove criteria include the following: (1) patients who voluntarily withdraw from this study or who are unable to operate for other reasons; (2) lung function is determined by the diagnostic criteria for ventilation dysfunction, such as guidelines from the American Thoracic Society and the European Respiratory Society [1, 2]; and (3) the expected value of FVC <80% was diagnosed as restrictive ventilation dysfunction, while FEV1/FVC <92% was diagnosed with obstructive ventilation disorder [2].

### 2.2. Research Methods

75 patients who met the abovementioned criteria after preliminary examination were collected after admission. Excluding 6 patients who did not agree to participate in the study and those who could not operate, a total of 69 patients (guardians) signed informed consent. Patients were randomly divided into traditional pulmonary training group and IS-enhanced pulmonary training group. The former is trained in traditional pulmonary training during perioperative period, while the latter is given training in traditional pulmonary and increased IS-enhanced pulmonary training. The lung function of the patient was measured at the time of admission to the hospital and after the completion of the training program lasting 1 week. It is confirmed that those who meet the surgical standards will undergo surgery after multidisciplinary consultation in the anesthetic department and other relevant disciplines. Those who do not meet the criteria continue to train in the above manner until the day before the operation. After surgery, it was determined whether to transit through the ICU as per the patient's cardiopulmonary function. The preoperative breathing training was continued after entering the ward. Chest CT flat sweep was used to assess the occurrence of lung complications 3 days after the surgery. Observation indicators are as follows: ① lung function at the time of admission and after one week of training; ② number of days of preoperative respiratory training; ③ postoperative respiratory complications; ④ time spent in the hospital after surgery; and ⑤ hospitalization costs and overall satisfaction. The research program was approved by the Ethics Committee of Drum Tower Hospital, affiliated with Nanjing University Medical College. Traditional pulmonary training is as follows: ① lip breathing: the patient aspirates with his nose and bulged the upper abdomen; holds the breath for 1-2 s after the lips slowly exhale through the mouth, while exhaling side count to 7 and exhale (inhale and exhale at 1 : 2); ② abdominal movement breathing: the patient places his hand on the abdomen and helps to contract the abdominal muscles when inhaling; takes a deep, slow breath, at which point the chest can be seen clearly lifted and the abdomen sinks; then, relaxes the abdomen and gently exhales breath; ③ muscle breathing: the nurse places his hands under the rib arch of the patient's abdomen and instructs the patient to inhale with his nose; the abdomen puffs outwards and then resists the nurse's hands when inhaling; holds the breath for 1-2 s to open the alveoli; uses mouth to slowly exhale gas when exhaling, and practices several times before the patient can practice on his own; ④ blow balloons: the patient takes a seat or a stand position and does the balloon movement; takes a deep breath, then holds the balloon, and blows all the gas from your lungs into the balloon until he cannot blow it out. The above method is professionally directed by the nurse to the patient. This is practiced 2 0 min per action, 3 times a day, and lung function tests are conducted again after 1 week. IS training is done with the Raventon Respiratory Trainer (Leventon S.A.U,SPIRO-BALL, L25913000 4000 ml). The patient sits on a chair and holds a lung capacity meter, moving the yellow indicator on the right side of the instrument up and down to the target milliliter (the initial training target is set at 70% of lung capacity based on lung function test results). Inhale deeply and evenly with mouth to keep the float in a raised state, and keep the small yellow ball in the smiley position for as long as possible and then exhale normally. Repeat 10 to 12 times per hour and 3 times a day for 1 hour, and repeat the lung function test after 1 week. The preoperative training regimen is continued on days 1 to 5 after surgery.

### 2.3. Lung Function Tests

Lung function testing is performed by fixed personnel using the German Jaeger lung function tester for patients after admission and one week after the completion of the exercise program. Observation indicators included vital capacity as a percentage of projected value (VC%), forced vital capacity as a percentage of predicted value (FVC%), percentage of forced expiratory volume in one second (FEV1%), forced expiratory volume in one second/forced vital capacity (FEV1/FVC, %), forced expiratory volume in one second/vital capacity (FEV1/VC,%), and maximal voluntary ventilation as a percentage of the estimated value (MVV%). Estimates are calculated based on the patient's age, weight, and finger spacing. The degree of lung impairment is based on current guidelines of the American Thoracic Society + European Respiratory Society and is diagnosed as restrictive ventilation dysfunction at an estimated FVC value < 80% [1, 2]. FEV1/FVC <92% is the diagnostic criteria for obstructive ventilation disorders.

### 2.4. Postoperative Respiratory Complications

On the third day after surgery, a routine chest CT scan was performed to observe whether there was atelectasis, lung infection, and pleural effusion.

### 2.5. Days Living in the Hospital before and after Surgery Were Recorded

Pain scores on days 1, 2, and 5 after surgery were recorded using a numeric rating scale (NRS) of pain.

### 2.6. Statistical Analysis

SPSS 23 statistical software is used for data processing and statistical analysis. Categorical variable data are expressed as counts or percentages, and continuous variables are expressed as means and standard deviations. Covariance analysis was used to compare lung function before and after one week of training. The intergroup outcome indexes between groups included lung complications and lung function, and *t*-test and chi-square test were used for comparison. All statistical tests were bilateral probability tests, and *p* < 0.05 was considered a statistically significant difference.

## 3. Results

A total of 75 patients who underwent scoliosis orthosis at the Spine Surgery Center of the Drum Tower Hospital affiliated to Nanjing University School of Medicine were admitted to the hospital and met the inclusion and exclusion criteria after preliminary examination from May 2019 to May 2021. In this study, a total of 6 patients were excluded based on the exclusion criteria. A total of 69 patients (guardians) signed informed consent forms by excluding 6 patients who did not agree to participate in this study and other reasons that they could not undergo surgery. It was randomly divided into the traditional pulmonary training group (*n* = 34) and IS-enhanced pulmonary training group (*n* = 35). Among them, 2 people voluntarily withdrew from the traditional pulmonary training group during the research process and 32 people finally completed the traditional training of the study. There were no significant differences in age (13.46 ± 6.36 vs. 15.66 ± 8.28, *p* = 0.233) and gender composition (male/female: 15/17 vs. 17/18, *p* = 0.89). The Cobb angle was 83.34 ± 7.93 in the traditional pulmonary training group and 83.97 ± 8.48 in the IS-enhanced pulmonary training group; there was no significant difference between the groups (*p* = 0.756). The basic lung function indicators of the two groups of patients are shown in Table 1. There was no significant difference in the degree and type of respiratory impairment (*p* = 0.621), and all presented with predominantly restrictive ventilation dysfunction.

There were no significant differences in the indicators of lung function upon admission between the two groups (*p* > 0.05). After one week of breathing training, both the traditional pulmonary training group and IS-enhanced pulmonary training group improved significantly(*p* > 0.01). Covariance analysis showed significant improvements in all other indicators except FEV1/VC (*p* = 0.031), indicating that the addition of stimulated spirometry training improved lung function more significantly than traditional training after 1 week of training. The improvement of other indicators varied significantly, indicating that added incentive spirometry training more significantly improved lung function after 1 week of training than conventional training (as shown in Table 2).

The incidence of postoperative pulmonary complications in both groups is shown in Table 3. No significant difference in the number of overall pulmonary complications between the two groups (*p* = 0.164) was found. However, the incidence of atelectasis in the traditional pulmonary training group (7/32, 21.9%) was much higher than that in the IS-enhanced pulmonary training group (1/35, 2.9%) (*p* = 0.043).

Comparison of hospital stay and postoperative pain scores between the two groups is shown in Table 4. The number of days living in the hospital before the traditional pulmonary training and IS-enhanced pulmonary training groups was 15.72 ± 9.65 and 11.34 ± 4.65, respectively. The difference was significant（*p* = 0.024）. The number of days living in the hospital after surgery was 15.13 ± 7.07 and 12.40 ± 2.50, with significant differences（*p* = 0.046）. The total length of days living in the hospital also varied significantly（*p* = 0.014）. There was no significant difference in pain scores on day 1 after surgery between the traditional pulmonary training and IS-enhanced pulmonary training groups (*p* = 0.085), while there was a statistical difference on day 2 postoperatively (*p* < 0.001), the difference was not significant on day 5 after surgery. In summary, there were significant differences between the two groups（*p* = 0.006).

## 4. Discussion

### 4.1. Impaired Respiratory Function Is an Important Complication of Scoliosis and Seriously Affects the Preoperative Safety of the Spinal Orthopedic Surgery

Scoliosis is the third largest “killer” affecting the health of children and adolescents with an incidence between 0.47% and 5.2% [3]. The incidence of scoliosis is about 1% in China. Scoliosis not only affects the appearance of the child but also causes pulmonary hypertension and impaired lung function [4–6]. In children with scoliosis, the elastic resistance of the lungs increases, the compliance decreases, the lung volume decreases, and the lung ventilation volume decreases due to the gradual change of bone structure and muscle structure [7]. Studies have shown that changes in lung function have a clear correlation with the degree of scoliosis. Patients with mild scoliosis deformity and Cobb angle below 60° have good compensatory lung function. Patients with moderate deformities and a Cobb angle of 60°–90° will have a significant decrease in VC, MVV, and FEV1, below 80% of the normal estimate. Patients with severe malformations and Cobb angles above 90° have VC, MVV, and FEV1 falling below 50% of normal estimates, with a minimum of only about 30%. The effect of scoliosis on respiratory function is also related to its etiology. Congenital scoliosis (CS) is particularly affecting respiratory function and can develop severe respiratory dysfunction. The effect on respiratory function is mainly manifested as a restrictive ventilation dysfunction regardless of the cause of scoliosis. The preoperative lung function test results of the enrolled patients in this study also met this feature.

Surgical orthopedics is the main means of treating scoliosis. However, lung injury, as one of the most common physiological dysfunctions caused by scoliosis, has become an important factor affecting surgical indications and perioperative safety. On the one hand, the presence of preoperative lung dysfunction can affect the safety of surgery; on the other hand, spinal orthopedics, in addition to its effects on the diaphragm and thoracic cage (anesthesia, wound pain, surgical stimulation, medications, and metabolic changes) has other causes of pulmonary complications. These combined factors can reduce lung volume and flow rate by 10% to 30%. In addition, VC and MVV are also reduced accordingly, and there is even a risk of developing postoperative pulmonary insufficiency or respiratory failure. The maximum preoperative ventilation volume has been reduced by 40% or less, and postoperative complications and the occurrence of ARDS may be greatly increased [8].

### 4.2. Lung Function Training Is an Important Part of the Perioperative Period of Spinal Orthopedic Surgery

Improving the compensatory capacity of lung function has always been the most important work in the perioperative period of scoliosis patients. Studies have shown that good respiratory exercises increase respiratory muscle strength by 35% to 55% and endurance by 19% to 55% [9]. Traditional preoperative respiratory rehabilitation training methods mainly include lip reduction breathing, balloon blowing, breathing exercises, etc. The resistance generated by the slow exhalation of the lip shrinking causes the isobaric pressure (2–5 cm H_2_O) point of the airway to move towards the distal end of the airway, which prevents the small airway from collapsing and narrowing during exhalation. This facilitated the discharge of alveolar air. The prolongation of the exhalation time is also conducive to the full discharge of gas in the lungs and prevents the airway from collapsing. Balloon blowing training can lengthen the exhalation time, slow down the airflow, and increase the internal pressure of the trachea to avoid premature collapse of the bronchi and small bronchi, thereby effectively eliminating residual gas in the lungs, improving the imbalance of ventilation/blood flow, reducing the dilution of functional residual gas to inhale the fresh air, increasing alveolar PCO2, and thus improving gas exchange and ventilation function. Respiratory exercises can increase the volume of lung ventilation, enhance the function of the respiratory muscles, reduce the residual air volume in the alveoli after forceful exhalation, and reduce the degree of alveolar expansion. Exercise can also improve breathing type, improve respiratory efficiency, increase muscle strength in the patient's limbs, and improve skeletal muscle dysfunction caused by chronic respiratory diseases.

### 4.3. IS-Enhanced Pulmonary Training and Its Role in Perioperative Lung Function Training

The lack of clear quality control indicators and the difficulty of self-assessment in the traditional respiratory function exercise process have a relationship with the patient's understanding and compliance, thus significantly reducing the efficiency of training. The Incentive Spirometer (IS) mimics sighing and yawning movements to help patients take an active approach to rhythmic deep breathing, increase tidal volume by promoting diaphragm respiration and alveolar opening, basal ventilation, penetrating pulmonary pressure, and in turn improve respiratory muscle status, promote airway secretion discharge, and assist to maximize lung expansion to prevent and reverse lung atelectasis [10–17]. An excitatory spirometer, also known as a sustained maximum inhalator, encourages patients to maximize inhalation and maintain >3 s by monitoring the flow or volume of breath. The excitatory spirometer is equipped with a display device that helps the healthcare provider guide the patient to the desired training effect, while the patient can also visually monitor the performance of their workout through the display [18].

Many studies have shown that IS can prevent the development of postoperative complications. Renault et al. [19] randomly divided 36 patients after coronary artery bypass surgery into deep breathing training group (*n* = 18) and IS group (*n* = 18). Although there were no significant differences in FVC and FEV between the two groups, the pulmonary complications in the IS group were significantly less than those in the deep breathing group. Alaparthi et al. [20] divided 260 patients undergoing laparoscopic abdominal surgery into four groups: diaphragmatic respiratory training group, volume IS group, flow IS group, and control group. FVC and FEV1 improved in all patients the day after surgery, while improvements were more prominent in the IS group. Rollin et al. [17] analyzed 84 patients and found that 5 of them had pneumonia in the routine care group, while none in the IS group had pneumonia. Koo and Hwang [21] divided 63 patients with epigastric surgery into control groups (*n* = 31) and IS groups (*n* = 32). Pulmonary complications occurred in 5 cases (16.1%) in the control group, compared with none in the IS group (*p* = 0.018).

However, Thomas and McIntosh [22] evaluated and compared the preventive effects of IS, IPPB, and deep breathing training in preventing pulmonary complications during epigastric surgery. The OR value for the development of pulmonary complications was 0.44 compared with patients without physical therapy, and the value was 0.43 for deep breathing training compared with patients without physical therapy. IS has included 46 studies in its role in preventing postoperative pulmonary complications, 35 without conclusions due to methodological defects, while 10 of 11 studies did not support the effect of IS. In the only study to support the outcome, IS, deep breathing training, and IPPB were equally effective in preventing lung complications after abdominal surgery. This evidence does not support that the use of IS after cardiac or abdominal surgery reduces the incidence of postoperative pulmonary complications. The findings suggest that IS deep breathing training after epigastric surgery is beneficial for preventing postoperative pulmonary complications, but there is no supportive evidence for differences between different treatments. The review by Carvalho et al. [23] included 30 studies (14 abdominal surgery, 13 cardiac surgery, and 3 thoracic surgery; total sample size is 3370). 5 studies (3 abdominal surgery, 1 cardiac surgery, and 1 thoracic surgery) compared the effects before and after IS interventions. No significant differences were found. The authors did not support the use of IS after surgery because no significant differences were found in the results. A Cochrane review [24] focused on coronary artery bypass surgery, including 7 studies of 592 patients; results were found to be superior to physiotherapy, positive pressure ventilation, active cycle breathing, or preoperative education. Patients with IS have poorer lung function and arterial support and no improvement in muscle strength compared with positive pressure ventilation. There is no evidence that IS reduces pulmonary complications or improves lung function after coronary artery bypass surgery. Another Cochrane review of epigastric surgery yielded similar results [25].

The subjects involved in the above studies are patients with basically normal preoperative lung function, and the subjects of this study are those whose lung function due to scoliosis has been moderately and severely impaired. The use of IS in the perioperative phase of scoliosis orthopedics has also been reported in the past, but most studies have included it as part of routine respiratory function training and have not independently quantified it. The results of this study show that the combination of stimulated lung capacity training based on traditional respiratory training can effectively improve the improvement of lung function indicators per unit time. After one week of training, the excitation spirometry training group had more significant improvements in the VC measured value/predicted value (%), FVC measured value/predicted value% (%), FEV1/FVC (%), FEV1/VC (%), FEV1/VC (%), and MVV measured/predicted value (%) compared with the traditional training group. In particular, the MVV improvement was more significant, suggesting that the addition of stimulated lung capacity training can more effectively improve the ventilation effect of the small airways.

Effective lung function training is also beneficial for patients' postoperative recovery. It can be seen that the improvement of respiratory function with the addition of IS training is more significant from this study, the preoperative respiratory function exercise time is significantly shortened, the postoperative pain score is significantly reduced, and the recovery time is shorter. In this study, the incidence of atelectasis alone in the postoperative complications of the IS-enhanced pulmonary training group was significantly improved, and the overall incidence of lung complications did not have a significant statistical change from that of the traditional pulmonary training group. Possible reasons are as follows: (1) the sample size of this study is still relatively small; (2) although the traditional training group did not improve lung function as much as the motivated lung capacity group within one week, its preoperative breathing training time was longer, and both groups of patients underwent surgery after the lung function training reached a certain standard. Therefore, the preoperative lung function training method had a limited impact on the occurrence of overall postoperative complications.

### 4.4. Attention for Is Training

The biggest advantage of IS is that it provides clear feedback during training, which can motivate patients to work hard to achieve the goals of each training session. Therefore, it is necessary to set a reasonable daily lung capacity training goal during use. The initial goal can be set according to lung function at the time of admission and then gradually increased according to the patient's condition. In addition, the completion of the goal still depends on patient compliance while an excitation spirometer provides visual feedback. Therefore, individualized training programs should be evaluated and implemented according to the specific situation of each patient, and the completion of the goals should be recorded every day and adjusted on time.

The clinical effect of IS is still controversial due to the lack of clinical evidence and the lack of clear standards for the use procedures and norms of IS, but the treatment and prevention effect of IS for pulmonary complications during the perioperative period is still generally agreed [26, 27]. The use of IS has four parameters: number of training sessions per day, target inhalation, number of breaths completed per session, and length of breath held after inhale, each of which varies greatly as described in the setup literature [28]. The motivational spirometer is not recommended alone and should be used in conjunction with other respiratory training measures according to the 2011 edition of the American Guidelines for Clinical Practice in Respiratory Care, including deep breathing, encouraging coughing, and early activity [29]. Reasonable anesthesia options are also important for preventing postoperative complications. Therefore, the guidance and implementation of traditional methods of respiratory function training should also not be relaxed when using motivated lung capacity training. In addition, IS includes two types: flow type and volume type, of which the volume type should be preferred in clinical use, especially for pediatric patients. The IS parameters used in this study are set by combining the characteristics of scoliosis patients and the previous experience of our center, and good results have been achieved in actual use.

### 4.5. Deficiencies of This Study

To more effectively observe the effects of IS in the perioperative period, the inclusion criteria for this study were set to patients with moderate and severe respiratory function at the time of admission and no severe respiratory failure, which greatly limited the sample size of the study and may have an impact on the outcome. Most patients with scoliosis orthopedic surgery have mild respiratory dysfunction, and a larger sample is needed for further study.

## 5. Conclusion

In this study, the preventive effect of pulmonary function training with IS on the improvement of pulmonary function and pulmonary complications was observed. IS is more conducive to the quantification of perioperative lung function training indicators and the standardization of processes, which is conducive to improving the efficiency of lung function training [30].

## Figures and Tables

**Table 1:** Basic information for both groups of patients.

Project	Traditional pulmonary training group (*n* = 32)	IS-enhanced pulmonary training group (*n* = 35)	*p* value
Age (year, *‾x* ± *s*)	13.47 ± 6.36	15.66 ± 8.28	0.233
BMI (kg/m^2^，*‾x* ± *s*）			
Gender (male/female)	15/17	17/18	0.890
Cobb angle (*‾x* ± *s*）	83.34 ± 7.93	83.97 ± 8.48	0.756
Pulmonary dysfunction (moderate/severe)	23/9	27/8	0.621
VC actual/predicted value (%)	48.69 ± 7.64	49.03 ± 7.19	.850
FVC actual/predicted value (%)	49.22 ± 8.08	49.55 ± 8.58	0.870
FEV1 actual/predicted value (%)	44.73 ± 8.13	44.68 ± 8.18	0.980
FEV1/FVC (%)	91.17 ± 9.21	90.22 ± 6.69	0.630
FEV1/VC (%)	91.99 ± 8.85	91.79 ± 6.07	0.912
MVV actual/predicted value (%)	52.01 ± 9.09	51.10 ± 9.89	0.696

VC: vital capacity; FC: forced vital capacity; FEV1: forced expiratory volume in one second; MVV: maximal voluntary ventilation.

**Table 2:** Analysis of covariance of lung function in two groups after completion of one-week training (analysis of covariance).

Covariant	Traditional pulmonary training group	IS-enhanced pulmonary training group	*p* value
VC actual value/predicted value (%)	48.69 ± 7.64	49.03 ± 7.19	0.85 (0.311)
First entered the hospital	60.46 ± 9.69	64.12 ± 7.68	0.09 (1.374)
After 1 week of training	11.77 ± 3.39	15.09 ± 3.39	0.006(8.116)
Difference *p* value	0.0001	0.0001	
FVC actual value/predicted value (%)	49.22 ± 8.08	49.55 ± 8.58	0.87 (0.005)
First entered the hospital	60.77 ± 9.62	65.96 ± 9.01	0.026(0.533)
After 1 week of training	11.55 ± 3.36	16.41 ± 2.91	0.0001(40.081)
Difference *p* value	0.0001	0.0001	
FEV1 actual value/predicted value (%)	44.73 ± 8.13	44.68 ± 8.18	0.98(0.848)
First entered the hospital	57.99 ± 7.92	63.98 ± 8.37	0.004(0.092)
After 1 week of training	13.27 ± 2.09	19.31 ± 2.85	0.0001(97.02)
Difference *p* value	0.0001	0.0001	
FEV1/FVC (%)	91.17 ± 9.21	90.22 ± 6.69	0.63(2.371)
First entered the hospital	94.72 ± 9.38	98.06 ± 7.20	0.105(1.46)
After 1 week of training	3.55 ± 7.16	7.84 ± 5.07	0.006(7.983)
Difference *p* value	0.009	0.0001	
FEV1/VC (%)	91.99 ± 8.85	91.79 ± 6.07	0.912(3.746)
First entered the hospital	95.65 ± 9.27	99.02 ± 6.53	0.088(3.336)
After 1 week of training	3.66 ± 7.49	7.23 ± 6.64	0.031(4.841)
Difference *p* value	0.009	0.0001	
MVV actual value/predicted value (%)	52.01 ± 9.09	51.10 ± 9.89	0.696(0.308)
First entered the hospital	59.12 ± 8.91	65.24 ± 12.59	0.026(3.331)
After 1 week of training	7.12 ± 3.44	14.14 ± 5.01	0.0001(44.759)
Difference *p* value	0.0001	0.0001	

**Table 3:** Postoperative pulmonary complications in two groups (chi-square test).

	Whole group（*n* = 67）	Traditional pulmonary training group（*n* = 32）	IS-enhanced pulmonary training group（*n* = 35）	*p* value
Pulmonary complications Yes	14 (20.9%)	9 (28.1%)	5 (14.3%)	0.164
No	53 (79.1%)	23 (71.9%)	30 (85.7%)	
Pneumonia yes	6 (9%)	4 (12.5%)	2 (5.7%)	0.587
No	61 (91%)	28 (87.5%)	33 (94.3%)	
Pleural effusion Yes	9 (13.4%)	5 (15.6%)	4 (11.4%)	0.885
No	58 (86.6%)	27 (84.4%)	31 (88.6%)	
Atelectasis Yes	18 (26.9%)	7 (21.9%)	1 (2.9%)	0.043
No	59 (88.1%)	25 (78.1%)	34 (97.1%)	

**Table 4:** Length of hospitalization, hospitalization cost, and pain score before and after surgery of the two groups of patients (*t*-test and chi-square test).

Whole group *n* = 67	Traditional pulmonary training group *n* = 32	IS-enhanced pulmonary training group *n* = 35	F value	*p* value
Length of preoperative hospital stay	15.72 ± 9.65	11.34 ± 4.65	12.96	0.024
Length of postoperative hospital stay	12.13 ± 7.07	9.37 ± 2.53	24.24	0.044
Total length in hospital	27.78 ± 14.65	20.66 ± 5.93	17.81	0.014
Postoperative pain score			8.107	.006
Day 1	2.09 ± 0.86	1.77 ± 0.65	3.060	0.085
Day 2	1.41 ± 0.71	0.77 ± 0.65	14.65	0.000
Day 5	0.28 ± 0.46	0.11 ± 0.32	3.026	0.087
Cost	21.12 ± 4.87	19.68 ± 2.69	7.49	0.148

## Data Availability

The datasets used and analyzed during the current study are available from the corresponding author upon reasonable request.
